# Prognostication in palliative radiotherapy—ProPaRT: Accuracy of prognostic scores

**DOI:** 10.3389/fonc.2022.918414

**Published:** 2022-08-16

**Authors:** Marco Maltoni, Emanuela Scarpi, Monia Dall’Agata, Simona Micheletti, Maria Caterina Pallotti, Martina Pieri, Marianna Ricci, Antonino Romeo, Maria Valentina Tenti, Luca Tontini, Romina Rossi

**Affiliations:** ^1^ Medical Oncology Unit, Department of Specialized, Experimental and Diagnostic Medicine (DIMES), University of Bologna, Bologna, Italy; ^2^ Unit of Biostatistics and Clinical Trials, IRCCS Istituto Romagnolo per lo Studio dei Tumori (IRST) “Dino Amadori”, Meldola, Italy; ^3^ Radiotherapy Unit, Istituto di Ricovero e Cura a Carattere Scientifico (IRCCS) Istituto Romagnolo per lo Studio dei Tumori (IRST) “Dino Amadori”, Meldola, Italy; ^4^ Palliative Care Unit, IRCCS Istituto Romagnolo per lo Studio dei Tumori (IRST) “Dino Amadori”, Meldola, Italy; ^5^ Palliative Care Unit, Azienda Unità Sanitaria Locale (AUSL) Romagna, Forlì, Italy

**Keywords:** outpatient palliative care, palliative radiotherapy, prognostication, aggressiveness of care, personalized palliative care

## Abstract

**Background:**

Prognostication can be used within a tailored decision-making process to achieve a more personalized approach to the care of patients with cancer. This prospective observational study evaluated the accuracy of the Palliative Prognostic score (PaP score) to predict survival in patients identified by oncologists as candidates for palliative radiotherapy (PRT). We also studied interrater variability for the clinical prediction of survival and PaP scores and assessed the accuracy of the Survival Prediction Score (SPS) and TEACHH score.

**Materials and methods:**

Consecutive patients were enrolled at first access to our Radiotherapy and Palliative Care Outpatient Clinic. The discriminating ability of the prognostic models was assessed using Harrell’s C index, and the corresponding 95% confidence intervals (95% CI) were obtained by bootstrapping.

**Results:**

In total, 255 patients with metastatic cancer were evaluated, and 123 (48.2%) were selected for PRT, all of whom completed treatment without interruption. Then, 10.6% of the irradiated patients who died underwent treatment within the last 30 days of life. The PaP score showed an accuracy of 74.8 (95% CI, 69.5–80.1) for radiation oncologist (RO) and 80.7 (95% CI, 75.9–85.5) for palliative care physician (PCP) in predicting 30-day survival. The accuracy of TEACHH was 76.1 (95% CI, 70.9–81.3) and 64.7 (95% CI, 58.8–70.6) for RO and PCP, respectively, and the accuracy of SPS was 70 (95% CI, 64.4–75.6) and 72.8 (95% CI, 67.3–78.3).

**Conclusion:**

Accurate prognostication can identify candidates for low-fraction PRT during the last days of life who are more likely to complete the planned treatment without interruption.

All the scores showed good discriminating capacity; the PaP had the higher accuracy, especially when used in a multidisciplinary way.

## Introduction

Prognostic evaluation is part of the overall assessment of cancer patients. Information on prognosis helps in clinical and therapeutic decision-making, patient and family counselling, and clinical research, facilitates the timely referral for palliative care (PC), and impacts the quality and costs of healthcare (1). A new approach to PC that takes into account the patients’ needs more than prognosis was recently proposed (2). However, the needs and prognostic factors in PC are not exclusive to each other but rather complementary and integrated in this care setting (3).

Within the areas of medical oncology and palliative care, it has been seen that an integrated, multiprofessional evaluation allows for a more complete assessment that takes into account different points of view, skills, and expertise (4, 5). Prognostic factors have further been combined to build prognostic scores or prognostic tools that can be used in the advanced phases of the disease (6–9). Issues such as needs assessment, prognostic evaluation, and multidisciplinary approaches have been proven useful for decision-making in the medical oncology/palliative care interface and have also been assessed by our group in a palliative radiotherapy (PRT) setting (10).

Around 50% of radiotherapy (RT) activities can be defined as PRT with a symptomatic or palliative aim. The decision-making process is complex and involves several issues, *i*.*e*., whether or not to perform RT, the choice of appropriate fractionation, the correct timing of RT to guarantee the relief or prevention of symptoms, and the best technique to use (11). Over the past few years, several prognostic factors have been developed for PRT. In particular, Chow et al. developed the SPS on 395 patients undergoing RT using six items weighted for their prognostic importance [primary cancer site, site of metastasis, Karnofsky performance status (KPS), fatigue, appetite, and shortness of breath]. The presence or absence of a number of these risk factors (NRF) was equally predictive and easier to manage. The median overall survival (OS) of the three groups evaluated with the NRF method was 62, 24, and 11 weeks (12). Thereafter, a simplified score (NRF) was built with only three factors: primary cancer site, site of metastasis and KPS. Three groups again were identified with a distinct survival of 15.0 *vs*. 6.5 *vs*. 2.3 months and a median OS of 4.9 months (13–15).

Krishnan et al. (16) developed another model (TEACHH model) to identify patients with short-term (<3 months) or long-term (>12 months) life expectancy within a population receiving PRT. The median survival of the entire group was 5.6 months. The score was built on factors that remained statistically significant at multivariate analysis: cancer type, ECOG PS, older age, number of prior palliative chemotherapy courses, hepatic metastases, and number of hospitalizations ≤3 months before PRT. The population was subdivided into three groups with different median survival. SPS NRF and TEACHH scores have been shown to be most effective for predicting survival at 3, 6, or 12 months and would appear to be less useful for predictions of short-term survival in an end-of-life setting (17).

The Palliative Prognostic Score (PaP score) was built and validated by our group (18, 19) and has been validated by independent groups (20–22) in a number of advanced cancer populations. PaP score consists of a “weighted” scoring system obtained with factors that remained statistically significant at multivariate analysis. The total scores ranged from 0 to 17.5 and assigned the patients to three different risk groups with a median survival of 10, 30, and 60 days (18, 19), showing a high accuracy at 88% (8).

In a study by Tayjasanant et al., the terms *advanced*, *end-of-life*, *terminal*, *end-stage*, and *dying* in cancer literature corresponded to a median survival of 114, 63, 42, 25, and 4 days, respectively (9). It has been reported that some scores are more useful in the advanced phase and others in the terminal phase of illness (6).

A recent study by Mojica-Marquez et al. (17) reported that, although both of these models provided accurate prognostication, they were more accurate in patients with a median survival of ≥3 months. In fact, in 505 patients with a median OS of 2.1 months, the TEACHH score correctly predicted life expectancy in 21.4% of cases, while the Chow model was accurate in 29.1%. The TEACHH method has also been used to select appropriate treatment to reduce the risk of 30-day mortality after PRT. In a study by Kain et al. (23), the 30-day mortality was 10% and was higher in patients in the TEACHH subgroups B/C (21% in C, 11% in B, and 2% in group A).

The study reported in the present paper, “Prognostication in palliative radiotherapy—ProPaRT” had the primary aim of evaluating the accuracy of the PaP score in a group of patients selected for PRT by oncologists. Working together with specialists from our Radiotherapy and Palliative Care Outpatient Clinic, this multidisciplinary team evaluated the 30-day prognostic accuracy to identify suitable candidates for PRT. The secondary endpoints of the study were as follows (1): to evaluate the interrater agreement between the clinical prediction of survival (CPS) and PaP score according to different professionals (radiation oncologists—RO and palliative care physicians—PCP) and (2) to assess and compare the accuracy of the SPS (PSM and NRF methods) and TEACHH (PSM and NRF methods) scores.

## Materials and methods

The organization of the integrated activities of the Radiotherapy and Palliative Care Outpatient Clinic has been described in detail elsewhere (10). The eligibility criteria for the present study were as follows: outpatients with advanced cancer (solid or hematologic tumors), ≥18 years old, and written informed consent. The patients were enrolled at their first access to the clinic, and the RO and PCP calculated all the prognostic scores simultaneously during the visit. A second appointment was scheduled for 1 month after the end of RT or 1 month after the first appointment for patients who were not amenable to RT. The patients were thereafter followed up for survival. All decisions regarding drug administration were taken by physicians and based on clinical judgment within the context of routine clinical practice, independently of the decision to include the patient in this study or not. Complete blood count data for this analysis were collected in the general laboratory of our hospital at a maximum of 7 days before or after the visit.

All the information needed to build the three prognostic scores (PaP, SPS, and TEACHH) were collected: age, KPS, CPS, dyspnea, anorexia, primary tumor site and type, location of all metastases, Eastern Cooperative Oncology Group Performance Status (ECOG PS), hospitalizations ≤3 months before the radiation consultation, and number of prior palliative chemotherapy and RT courses.

The PaP score was obtained from a Weibull multivariate regression model including six variables (KPS, CPS, anorexia, dyspnea, total white blood count, and lymphocyte percentage) chosen after a backward selection procedure from a set of 34 biological and clinical factors (18, 19). Each variable was allotted a “partial score” related to the size of the regression coefficient. The sum of the partial scores produced the PaP score. The total scores range between 0 and 17.5 and assigned the patients to one of three different risk groups according to a 30-day survival probability: group A, >70%; group B, 30–70%; and group C, <30% (**Supplementary Table S1**).

The SPS (12–15) was obtained in two ways. The first method (PSM) consisted in assigning a partial score on the basis of the prognostic “weight” of a single factor to each of the factors included (primary cancer site, site of metastases, and KPS) and then adding them together. The second method (NRF) consisted in grouping patients according to the total number of risk factors that they possessed. The three risk factors were non-breast cancer, sites of metastasis other than bone, and KPS ≤60 (**Supplementary Table S2**) (13).

The TEACHH model (16) divided the patients receiving PRT into three distinct life expectancy groups based on both the PSM and the NRF methods. For the PSM method, the partial scores for each variable were summed up to calculate a total PSM score for each patient. Each patient’s NRF score was based on the sum of those predictors present. The PSM and NRF methods’ scores were then used to classify the patients into three groups aimed at identifying those with the poorest (≤3 months) and best (>1 year) life expectancy (**Supplementary Table S3**).

The study was approved by the Area Vasta Romagna Ethics Committee (code L2P1517 of May 17, 2017) and performed with the 1964 Helsinki Declaration and its later amendments and with Good Clinical Practice guidelines. Written informed consent was obtained from all individual participants included in the study. No identifiable human data were included in the manuscript.

### Statistical analysis

Continuous variables were summarized by descriptive statistics (number of cases, mean, standard deviation, median, minimum, and maximum) and categorical variables using counts of patients and percentages. Overall survival was defined as the time from the date of enrollment in the study to the date of death from any cause or the date of the last available information. Survival curves were estimated using the product-limit method of Kaplan–Meier and compared by log-rank test. The discriminating ability of the prognostic models was assessed using Harrell’s C index, and the corresponding 95% confidence intervals (95% CI) were obtained by bootstrapping. Overall accuracy, sensitivity, specificity, positive predictive value, negative predictive value, and relative 95% CI were calculated at the 30th day of follow-up. The inter-rater agreement of the CPS and the PaP scores between the RO and PCP was measured with the Kappa statistic: kappas over 0.75—excellent, 0.40 to 0.75—fair to good, and below 0.40—poor (24). Assuming an accuracy level of 88% and a precision level of 4%, with an estimated type I error of 5% type, and using two-tailed test, a total recruitment of 254 patients was needed for the study. Statistical analyses were performed using SAS software, version 9.4 (SAS Inst., Cary, NC, USA).

## Results

This prospective, observational study enrolled 255 patients with metastatic cancer referred from medical oncologists at the Radiotherapy and Palliative Care Outpatient Clinic from August 2017 to April 2020. The patients were evaluated jointly by a RO and PCP. The patients’ characteristics are reported in **Table 1**. Median age was 70 years (interquartile range, 60–77), and 141 (55.3%) were male patients. Lung cancer was the most frequent primary tumor (30.9%), followed by breast (22.3%) and tumors of the urogenitary tract (13.7%). Bone metastases were present in 72.9%, and there was lymph node involvement in 44.3%. Sixty-six (25.9%) patients had one site of metastatic disease at the first RaP visit, 95 (37.3%) had two sites, and 94 (36.8%) had three or more. PRT was indicated in 123 patients (48.2%) of the 255 patients at the first visit.

**Table 1:** Main clinical–biological charactestistics of 255 patients.

Variables	Number	%
**Median age, years (range; IQR)**	70 (38–99; 60–77)
≤60	67	26.3
>60	188	73.7
**Gender**		
Male	141	55.3
Female	114	44.7
**Primary tumor site**		
Lung	79	30.9
Breast	57	22.3
Prostate	27	10.6
Urogenitary tract (not prostate)	35	13.7
Gastrointestinal tract	27	10.6
Others	34	13.3
**Metastatic sites**		
Bone	186	72.9
Lymph nodes	113	44.3
Lung	76	29.8
CNS	55	21.6
Liver	42	16.5
Soft tissue	13	5.1
Locally advanced disease	36	14.1
Other	46	18.0
**Number of metastatic sites**		
1	66	25.9
2	95	37.3
3	71	27.8
4	17	6.7
5	6	2.3

IQR, interquartile range; CNS, central nervous system.

Seventy-six (61.8%) patients selected for PRT underwent a single fraction schedule, 43 (35.0%) had two to five fractions, one (0.8%) had 10 fractions, and 3 (2.4%) had >10 fractions (**Table 2**). All irradiated patients completed the treatment as planned. There was an average interval of 40.6 (standard deviation 194.5) days between the last dose of chemotherapy and the visit in the Radiotherapy and Palliative Care Outpatient Clinic (median, 9 days; range, 0–2,624; interquartile range, 5–22). At the time of analysis, 83 (67.5%) irradiated patients had died: 26 (31.3%) underwent RT in the last 60 days of life, of whom 13 (15.6%) had it in the last 30 days. None of the patients had RT in the last 10 days of life. Eighteen patients died within 30 days of the first RaP visit, but only three were treated with PRT, indicating a 30-day survival from the first visit to death of 2.4%. In treated patients, 13 died within 30 days, representing a 30-day mortality rate of 10.6%. With regard the OS of the entire group, median follow-up was 484 days (range, 9–1,064), and median OS was 250 days (95% CI, 200–342). The median OS in the 123 patients undergoing RT was 274 (95% CI, 190–416) days, and it was 234 (95% CI, 186–376) days in those (*n* = 132) who did not receive RT (*p* = 0.702).

**Table 2:** Characteristics of palliative radiotherapy in 123 patients.

Variables	Number	%
**Irradiated sites**		
Bone	88	71.6
CNS	18	14.6
Visceral	6	4.9
Lymph nodes	5	4.1
Soft tissue	4	3.2
Other	2	1.6
**Number of fractions**		
1	76	61.8
2–5	43	35.0
10	1	0.8
>10	3	2.4

CNS, central nervous system.

**Figure 1:**
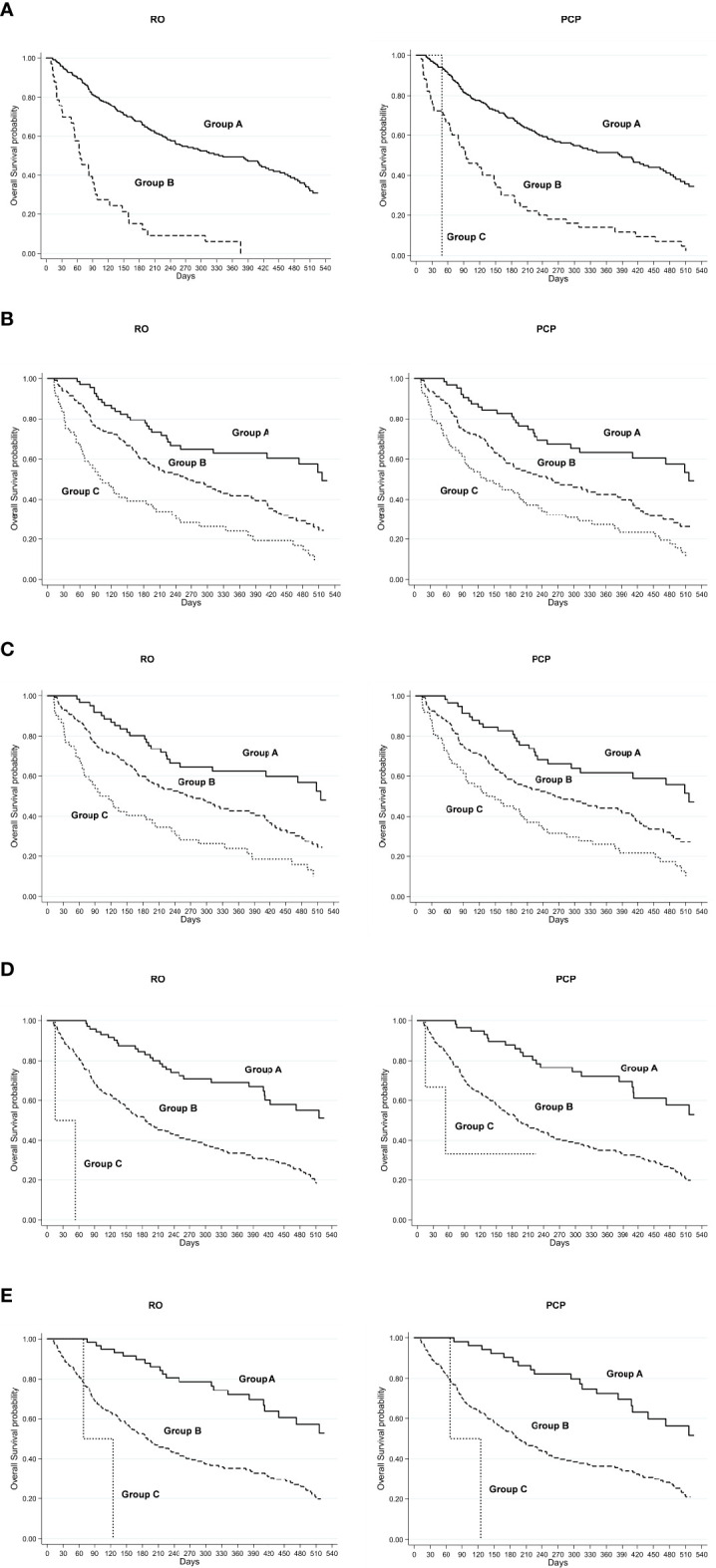
RO, radiation oncologist; PCP, palliative care physician; Kaplan–Meier overall survival curve for low-risk (group A), intermediate-risk (group B), and high-risk (group C) groups defined by **(A)** PaP score, **(B)** SPS score (PSM), **(C)** SPS score (NRF), **(D)** TEACHH score (PSM), and **(E)** TEACHH score (NRF).

Each prognostic score was calculated separately by both the RO and PCP. The univariate analysis of OS according to prognostic scores and evaluations of RO/PCP is reported in **Table 3**. According to the scores calculated by RO, 222 (87%) patients were classified in PaP score class A, 33 (13%) in class B, and 0 in class C, with a median OS of 334 days for class A and 65 days for class B. The TEACHH score (PSM) also subdivided the population into two groups, with 28.2% of patients in class A (median OS not reached), 71% in class B (median OS, 186 days), and only 0.8% in class C (median OS, 34 days). The SPS score (PSM) showed 26.6% of patients in class A (median OS, 516 days), 51.4% in class B (median OS, 263 days), and 22% in class C (median OS, 101 days). Similar results were obtained for the scores calculated by NRF or by the PCP. All prognostic scores identified groups with different prognoses (*p* < 0.0001) (**Figures 1A–E**).

**Table 3:** Univariate analysis of overall survival (OS) according to scores and different professionals.

	RO				PCP			
Risk groups	Number of patients	Number of events	Median OS (days)(95% CI)	30-day OS, %(95% CI)	Number of patients	Number of events	Median OS (days)(95% CI)	30-day OS, % (95% CI)
**PaP score (PSM)**
A (0–5.5)	222	133	334 (249–431)	96 (93–99)	203	116	385 (263–468)	97 (94–99)
B (5.6–11.0)	33	32	65 (48–93)	73 (58–88)	51	48	95 (63–148)	76 (64–88)
C (11.1–17.5)	0	–	–	–	1	1	48 (-)	100
*p*-value			<0.0001				<0.0001	
C-index (95% CI)			0.81 (0.69–0.93)				0.82 (0.72–0.92)	
**SPS (PSM)**
A (0–4)	68	29	516 (311-nr)	100	64	27	516 (311-nr)	100
B (5)	131	88	263 (185–382)	94 (90–98)	124	81	259 (168–389)	94 (89–98)
C (6–8)	56	48	101 (65–185)	82 (72–92)	67	57	134 (85–209)	85 (77–94)
*p*-value			<0.0001				<0.0001	
C-index (95% CI)			0.73 (0.64–0.82)				0.70 (0.60–0.80)	
**TEACHH (PSM)**
A (0–4)	72	29	nr	100	58	21	nr	100
B (5–15)	181	134	186 (147–240)	91 (86–95)	194	142	190 (150–249)	91 (87–95)
C (16–22)	2	2	34 (15-nr)	50 (0–100)	3	2	53 (15-nr)	67 (13–100)
*p*-value			<0.0001				<0.0001	
C-index (95% CI)			0.81 (0.74–0.88)				0.76 (0.67–0.84)	
**SPS (NRF)**
I (0–1)	61	27	516 (311-nr)	100	58	26	516 (294-nr)	100
II (2)	142	94	263 (185–389)	93 (89–97)	135	86	263 (170–409)	93 (88–97)
III (3)	52	44	107 (65–197)	85 (75–94)	62	53	141 (85–209)	87 (79–95)
*p*-value			<0.0001				<0.0001	
C-index (95% CI)			0.69 (0.59–0.78)				0.66 (0.56–0.76)	
**TEACHH (NRF)**								
A (0–1)	59	22	575 (411-nr)	100	52	19	nr	100
B (2–4)	194	141	190 (149–240)	91 (87–95)	201	144	196 (156–249)	91 (87–95)
C (5–6)	2	2	96 (68-nr)	100	2	2	96 (68-nr)	100
*p*-value			<0.0001				<0.0001	
C-index (95% CI)			0.80 (0.72–0.89)				0.77 (0.67–0.86)	

nr, not reached; RO, radiation oncologist; PCP, palliative care physician; PSM, partial score method; NRF, number of risk factors; CI, confidence interval.

The PaP score proved to be the best at discriminating patient prognosis as the median OS and 30-day survival probability were more in line with those of the risk group in which the patients were categorized. The SPS score showed a poorer performance in discriminating patients with better or worse prognosis. The TEACHH score (evaluated with PSM) had results similar to those of the PaP score.

The PaP score showed an accuracy of 74.8 (95% CI, 69.5–80.1) for RO and 80.7 (95% CI, 75.9–85.5) for PCP in predicting 30-day survival. The other scores, calculated after selecting the best cutoff, are detailed in **Table 4**.

**Table 4:** Accuracy of scores.

Score	Cutoff^a^	% sensitivity (95% CI)	% specificity (95% CI)	% PPV (95% CI)	% NPV (95% CI)	% accuracy (95% CI)
**RO**						
PaP score	5	70.6 (48.9–92.3)	74.7 (69.2–80.2)	25.6 (14.3–36.9)	97.2 (94.9–99.5)	74.8 (69.5–80.1)
SPS score (PSM)	7	41.2 (17.8–64.6)	76.8 (71.4–82.2)	14.9 (4.7–25.1)	95.2 (94.3–96.1)	70.0 (64.4–75.6)
SPS score (NRF)	2	41.2 (17.8–64.6)	81.1 (76.0–86.0)	13.5 (4.2–18.5)	95.1 (92.3–97.9)	68.7 (63.0–74.4)
TEACHH score (PSM)	10	82.4 (64.3–100)	75.8 (70.4–81.2)	19.4 (10.3–28.5)	98.4 (96.6–100)	76.1 (70.9–81.3)
TEACHH score (NRF)	3	88.9 (82.9–94.9)	59.7 (53.5–65.9)	25.8 (10.4–41.2)	96.0 (94.1–97.4)	61.8 (55.8–67.8)
**PCP**						
PaP score	5	76.5 (56.3–96.7)	81.4 (76.6–86.4)	21.0 (1.6–40.4)	97.9 (96.1–99.7)	80.7 (75.9–85.5)
SPS score (PSM)	7	41.2 (17.8–64.6)	81.0 (76.0–86.0)	12.5 (3.8–21.2)	95.0 (92.2–97.8)	72.8 (67.3–78.3)
SPS score (NRF)	2	41.2 (17.8–64.6)	76.9 (71.6–82.3)	11.3 (3.4–19.2)	94.8 (91.7–97.9)	66.2 (60.4–72.0)
TEACHH score (PSM)	10	76.5 (56.3–96.7)	63.9 (57.8–70.0)	13.1 (4.0–22.2)	97.4 (95.2–99.6)	64.7 (58.8–70.6)
TEACHH score (NRF)	3	88.9 (82.9–94.9)	53.8 (47.5–60.1)	20.6 (7.0–34.2)	95.5 (93.1–97.0)	56.3 (50.3–62.5)

RO, radiation oncologist; PCP, palliative care physician; PSM, partial score method; NRF, number of risk factors; PPV, positive predictive value; NPV, negative predictive value; CI, confidence interval.

^a^We chose to show the best performance cutoff for each score.

The C index of the PaP score was 0.81 (95% CI, 0.69–0.93) and 0.82 (95% CI, 0.72–0.92) for RO and PCP, respectively, while that of the CPS was 0.711 (95% CI, 0.57–0.85) and 0.79 (95% CI, 0.64–0.88), respectively. Considering only irradiated patients (the TEACHH score was originally built only on the group of patients undergoing RT), the accuracy was as follows: PaP score: 70.7 (95% CI, 65.1–76.3) and 80.5 (95% CI, 75.6–85.4), SPS-PSM: 74.0 (95% CI, 68.6–79.4) and 78.9 (95% CI, 73.9–83-9), SPS-NRF method: 26.8 (95% CI, 21.4–32.2) and 25.2 (95% CI, 19.9–30.5), TEACHH-SPM: 69.1 (95% CI, 63.4–74.8) and 59.3 (95% CI, 53.3–65.3), and TEACHH-NRF: 49.6 (95% CI, 43.7–55.7) and 46.3 (95% CI, 40.2–52.4).

The interrater agreement between RO and PCP was 0.51 (95% CI, 0.42–0.59) for CPS and 0.75 (95% CI, 0.70–0.79) for the PaP score. The interrater agreement of the scores between RO and PCP using SPS-PSM was 0.87 (95% CI, 0.82–0.92); this was 0.88 (95% CI, 0.83–0.93) for SPS-NRF, 0.86 (95% CI, 0.82–0.90) for TEACCHH-PSM, and 0.90 (95% CI, 0.87–0.94) for the TEACHH-NRF scores. These agreements were higher than that of the PaP score because of the presence of a larger number of objective factors.

Both SPS and TEACHH predictive capacity, calculated using the PSM and NRF methods, did not differ (data not shown). It follows that, given the same accuracy, the simplest method (NRF) is the best one to use.

PaP score accuracy was also compared with that of CPS alone. The C index of PaP score was 0.81 (95% CI, 0.69–0.93) for RO and 0.82 (95% CI, 0.72–0.92) for PCP. The PaP score had a higher C index than that of CPS alone (0.71, 95% CI: 0.57–0.85 for RO and 0.76, 95% CI: 0.64–0.88 for PCP) and than that of the PaP score without CPS (0.78, 95% CI: 0.67–0.89 for RO and 0.78, 95% CI: 0.67–0.89 for PCP).

## Discussion

Prognosis in PRT should be systematically evaluated to decide whether or not to pursue the recommended treatment (and if so, with which schedule). We chose to focus on a 30-day survival prediction because this cutoff seemed the most suitable to manage patients assigned to PRT at the end of life. A too-optimistic prediction of survival can have negative iatrogenic effects and an unfavorable impact on the indicators of poor quality of care such as an increase in the request for futile aggressive treatments, late referral to palliative care settings, and a higher percentage of deaths in hospital (sometimes in the intensive care unit) (25–27).

Efforts have been made using different methods to improve CPS performance, *e*.*g*., in a temporal way, in a probabilistic way, and using the surprise question (28–31). Nonetheless, CPS alone continues to show limited accuracy, often overestimating the survival lifespan. CPS has also been tested in the PRT setting and shown insufficient prognostic accuracy. Chow et al. reported on 739 patients (median survival, 15.9 weeks) for whom six ROs calculated estimates of survival. The mean difference between actual survival and CPS was 12.3 weeks, indicating an inaccurate prediction of survival in an optimistic sense (32). Benson reported that, out of 877 predictions by 22 ROs, only 39.7% were accurate, with 26.5 underestimations and 33.9% overestimations. The estimates were considered accurate when the actual OS was within the prediction category (0–6 months, >6–12 months, >12–24 months, and >24 months). Using this definition of accuracy, there was an overall 60.3% of inaccurate predictions, albeit with a less systematic overprediction than that usually reported in the literature. Predictions were most accurate for lower KPS (33).

In a study by Razvi et al. (34), CPS used alone did not perform better, with an overestimation in 78.5% of cases and a survival overestimation of 19.0 weeks on average. The inaccuracy was even greater than that of a similar but older study (32) in which the difference between predicted and actual survival was 12.3 weeks. Sborov et al. reported that 22% of clinical predictions of survival by ROs were incorrect in an optimistic sense. The optimistic prediction was related to aggressive end-of-life in the last 30 days of life as an additional operational metric (35).

Other authors have described futile behavior in end-of-life care. In a SEER study by Guadagnolo et al., 15,287 patients received RT in the last month of life. Of these, 2,721 (17.8%) received more than 10 days of treatment. Almost one in five patients who underwent RT in their final 30 days of life spent more than 10 of those days receiving treatment (36). From 2000 to 2009, there was also an increase in the number of patients treated in the last 30 days of life with three-dimensional RT with respect to two-dimensional RT (from 27.2 to 58.5%) and with intensity-modulated RT (from 0 to 6%). There was no evidence of improved quality of life or OS from this increase in treatment intensity (37).

A systematic review by Park et al. showed that PRT was performed in the last 30 days of life in 5–10% of patients who died of cancer and in 9.0–15.3% of those who underwent PRT. Single fractions were used in 0 to 59% of patients, while the majority received 30 Gy in 10 fractions (36 to 100%), with a high rate of incomplete treatments (53–83%). This suggests that shorter or single-fraction regimens are more appropriate, especially in patients with poor performance status (38). A study by Gripp et al. reported on 33 patients who died within 30 days from RT. Only 16% of the survival estimates made by ROs were correct, suggesting that RT was not adequately tailored in this population. Only 58% of patients completed RT, indicating that just under half spent 60% or more of their remaining life undergoing treatment (39).

In medical oncology, many tools have been tested, but only a few have been validated by independent researchers (40, 41)

In the present study, which is focused on 30-day survival prediction, the PaP score calculated by both the RO and PCP showed good accuracy and performed a little better than the other scores. The integrated RO and PCP Outpatient Clinic obtained a 30-day mortality rate after PRT of 8.9%, which was lower than the rates reported in other studies (42–44) and lower than the 20% recommended by the Royal College of Radiologists. Moreover, there were no interruptions in PRT (single fraction in 61.8% and five fractions in 35%), and 51.8% of patients were spared from futile RT, with an overall 30-day mortality from the time of first access to the outpatient clinic of 2.4%. Of the 123 treated patients, 13 (10.6%) were treated in the last 30 days, and none died in the last 10 days of life The PSM- and NRF-based SPS and the SPS- and NRF-based TEACHH had a higher interrater concordance than the PaP score as they are built on more objective factors, but with a lower level of accuracy. However, the interrater concordance of PaP was higher than that of CPS alone as it is corrected by objective factors.

Our study had a number of limitations. Given that it was a monocenter study, the results were limited to a single population. Moreover, it was performed in an outpatient clinic in which ROs, PCPs, and nursing staff worked as a team. Finally, as the PaP score is more accurate in the final trajectory of the disease, the overall population was not divided into three balanced groups.

## Conclusions

Our prospective, observational study had a sharply focused aim and a patient sample coherent with the needs of the study, *i*.*e*., to understand whether the PaP score maintains its predictive capacity in terminally ill cancer patients undergoing PRT. This capacity was confirmed by our results. The interrater variability of the score was good but slightly less than that of the other scores that had more objective items. Although all the scores showed good discriminating capacity, the PaP had the higher accuracy, especially when used in a multidisciplinary way.

## Data availability statement

The original contributions presented in the study are included in the artile/**Supplementary Material** (**Table S4**).

## Ethics statement

This study was reviewed and approved by Area Vasta Romagna Ethics Committee. The patients/participants provided their written informed consent to participate in this study.

## Author contributions

Conception and design: MM, RR, and ES. Collection and assembly of data: MDA and RR. Data analysis and interpretation: MM, ES, and RR. Manuscript writing: all authors. Final approval of the manuscript: all authors. All authors contributed to the article and approved the submitted version.

## Funding

This work was partly supported thanks to the contribution of Ricerca Corrente by the Italian Ministry of Health within the research line "Appropriateness, outcomes, drug value and organizational models for the continuity of diagnostic-therapeutic pathways in oncology" (L2P1517).

## Conflict of interest

The authors declare that the research was conducted in the absence of any commercial or financial relationships that could be construed as a potential conflict of interest.

## Publisher’s note

All claims expressed in this article are solely those of the authors and do not necessarily represent those of their affiliated organizations, or those of the publisher, the editors and the reviewers. Any product that may be evaluated in this article, or claim that may be made by its manufacturer, is not guaranteed or endorsed by the publisher.

## Abbreviations

TEACHH, Type of cancer, ECOG PS, age, prior palliative chemotherapy, prior hospitalizations and hepatic metastases; CPS, clinical prediction of survival; KPS, Karnofsky performance status; NRF, number of risk factors; OS, overall survival; PaP score, palliative prognostic score; PC, palliative care; PCP, palliative care physician; PRT, palliative radiotherapy; PSM, partial score method; RO, radiation oncologist; RT, radiotherapy; SPS, survival prediction score; WBC, white blood count.
