# Supplementary material for: Prognostication in palliative radiotherapy—ProPaRT: Accuracy of prognostic scores
Source: Front Oncol. 2022 Aug 16;12:918414. doi: 10.3389/fonc.2022.918414 (PMC9425085; doi:10.3389/fonc.2022.918414)
Supplement: Supplementary file 1 [file Table_1.docx]

**TABLE 1S | Palliative Prognostic Score: PaP Score**

|  |  | **Partial score** |
| --- | --- | --- |
| **Dyspnea** | No | 0 |
|  | Yes | 1 |
| **Anorexia** | No | 0 |
|  | Yes | 1.5 |
| **KPS** | ≥50 | 0 |
|  | 30-40 | 0 |
|  | 10-20 | 2.5 |
| **CPS (weeks)** | >12 | 0 |
|  | 11-12 | 2.0 |
|  | 9-10 | 2.5 |
|  | 7-8 | 2.5 |
|  | 5-6 | 4.5 |
|  | 3-4 | 6.0 |
|  | 1-2 | 8.5 |
| **Total WBC** | Normal (4800-8500 cell/mm^3^) | 0 |
|  | High (8501-11000 cell/mm^3^) | 0.5 |
|  | Very high (>11000 cell/mm^3^) | 1.5 |
| **Lymphocyte percentage** | Normal (20.0-40.0%) | 0 |
|  | Low (12.0-19.9%) | 1.0 |
|  | Very low (0-11.9%) | 2.5 |

*KPS*, Karnofsky Performance Status; *CPS*, Clinical Prediction of Survival; *WBC*, White Blood Count.

| **PaP Score** |  |
| --- | --- |
| **Risk groups** | **Total PaP score** |
| A (30-day survival probability >70%) | 0.0-5.5 |
| B (30-day survival probability 30-70%) | 5.6-11.0 |
| C (30-day survival probability <30%) | 11.1-17.5 |
