# Supplementary material for: Prognostication in palliative radiotherapy—ProPaRT: Accuracy of prognostic scores
Source: Front Oncol. 2022 Aug 16;12:918414. doi: 10.3389/fonc.2022.918414 (PMC9425085; doi:10.3389/fonc.2022.918414)
Supplement: Supplementary file 2 [file Table_2.docx]

**TABLE 2S | Survival Prediction Score**

|  |  | **PSM** |
| --- | --- | --- |
| **Site of primary cancer** | Breast | 0 |
|  | Prostate | 2 |
|  | Lung | 3 |
|  | Other | 3 |
| **Site of metastasis** | Bone only | 0 |
|  | Other | 2 |
| **KPS** | >60 | 0 |
|  | ≤60 | 3 |

*KPS*, Karnofsky Performance Status; *PSM*, Partial Score Method; *SPS*, Survival Prediction Score; *NRF*, Number of Risk Factors.

| **SPS score** | **PSM** | **NRF** |
| --- | --- | --- |
| **Risk groups** | **Total score** | **Total score** |
| A – I | 0-4 | 0-1 |
| B – II | 5 | 2 |
| C- III | 6-8 | 3 |
