# Supplementary material for: Prognostication in palliative radiotherapy—ProPaRT: Accuracy of prognostic scores
Source: Front Oncol. 2022 Aug 16;12:918414. doi: 10.3389/fonc.2022.918414 (PMC9425085; doi:10.3389/fonc.2022.918414)
Supplement: Supplementary file 3 [file Table_3.docx]

**TABLE 3S | Type of cancer, ECOG PS, age, prior palliative chemotherapy, prior hospitalizations and hepatic metastases: TEACHH score**

|  |  | **PSM** | **NRF** |
| --- | --- | --- | --- |
| **Site of primary cancer** | Breast | 0 | 0 |
|  | Prostate | 0 | 0 |
|  | Lung | 5 | 1 |
|  | Others | 2 | 1 |
| **ECOG PS** | 0-1 | 0 | 0 |
|  | 2 | 4 | 1 |
|  | 3-4 | 6 | 1 |
| **Liver metastasis** | No | 0 | 0 |
|  | Yes | 4 | 1 |
| **No. of prior palliative chemotherapy courses** | 0-2 | 0 | 0 |
|  | >2 | 3 | 1 |
| **Age (years) at treatment** | ≤60 | 0 | 0 |
|  | >60 | 2 | 1 |
| **Hospitalizations in previous 3 months** | No | 0 | 0 |
|  | Yes | 2 | 1 |

*ECOG PS*, Eastern Cooperative Oncology Group Performance Status; *PSM*, Partial Score Method; *NRF*, Number of Risk Factors.

| **TEACHH score** | **PSM** | **NRF** |
| --- | --- | --- |
| **Risk groups** | **Total score** | **Total score** |
| A | 0-4 | 0-1 |
| B | 5-15 | 2-4 |
| C | 16-22 | 5-6 |
